# Supplementary material for: StrIDR: a database of intrinsically disordered regions of proteins with experimentally resolved structures
Source: bioRxiv. 2024 Aug 26:2024.08.22.609111. Preprint. [Version 1] doi: 10.1101/2024.08.22.609111 (PMC11382991; doi:10.1101/2024.08.22.609111)
Supplement: Supplement 1 [file media-1.pdf]

Supplementary Information

StrIDR: a database of intrinsically disordered regions of proteins with experimentally resolved structures

Kartik Majila[1] and Shruthi Viswanath[1,+]

[1]National Centre for Biological Sciences, Tata Institute of Fundamental Research, Bangalore, India 560065.

[+]Corresponding author. Email: shruthiv@ncbs.res.in (SV)

Supplementary Figures

Figure S1: Distribution of disordered residues in the StrIDR database. Number of disordered residues per PDB entry included in StrIDR. For ease of visualization, the maximum number of disordered residues per PDB has been capped to 1000.

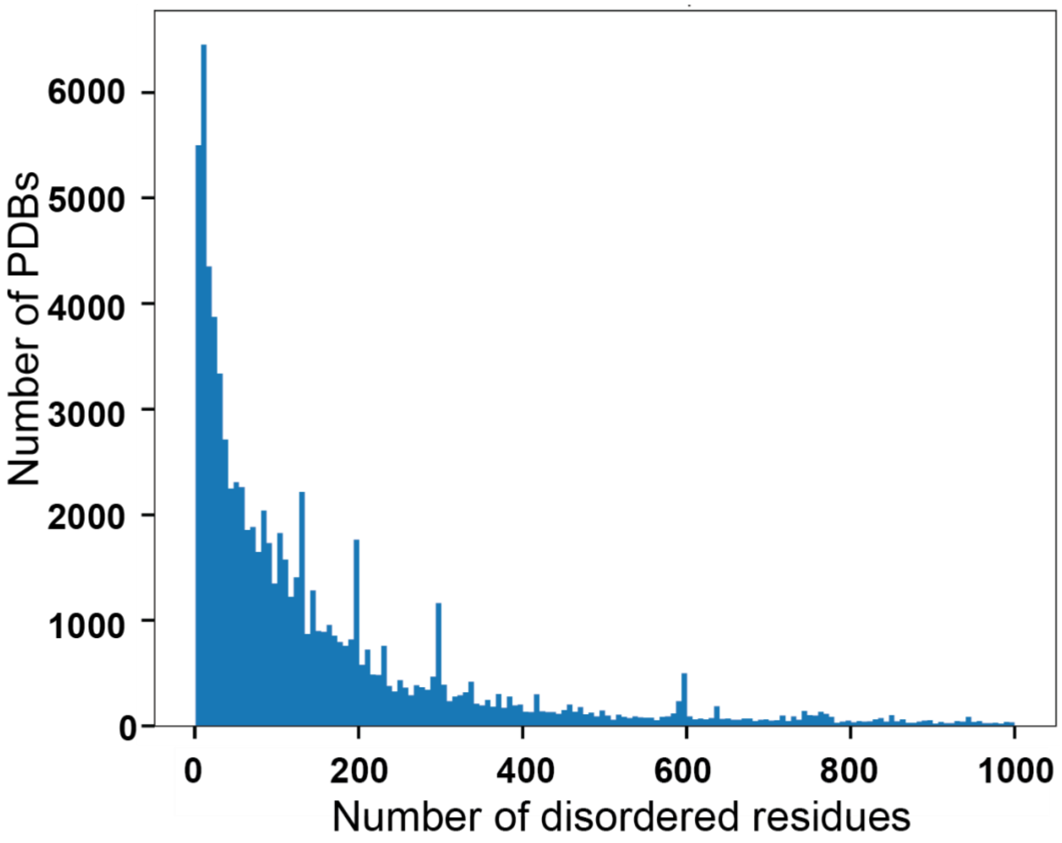

Supplementary tables

Table 1. Database Sources. Disordered protein databases and datasets used for StrIDR database creation. We obtain UniProt entries from all the databases. The acronyms used are as follows: IDR: intrinsically disordered region of a protein, DOR: disorder-to-order, DDR: disorder-to-disorder.

Database	Description	URL	Reference	Total UniProt IDs
DIBS	Database of complexes between an IDR and an ordered partner protein; the IDRs undergo a DOR transition	https://dibs.enzim.ttk.mta.hu/	(Schad et al., 2018)	772
MFIB	Database of complexes between IDRs; the IDRs undergo a DOR transition	https://mfib.enzim.ttk.mta.hu/	(Fichó et al., 2017)	1329
FuzDB	Dataset of complexes containing IDRs; the IDR undergoes a DDR transition	https://fuzdb.org/	(Miskei et al., 2017)	404
DisProt	Manually curated repository for structure and function of experimentally verified IDRs.	https://disprot.org	(Aspromonte et al., 2024) (Piovesan et al., 2017) (Vucetic et al., 2005)	3064
IDEAL	Database for experimentally verified IDRs with focus on Protean segments.	https://www.ideal-db.org/	(Fukuchi et al., 2014)<br><br>(Fukuchi et al., 2012)	1015
MobiDB	Database for predictions and annotations on protein disorder and mobility	https://mobidb.bio.unipd.it/	(Piovesan et al., 2023)<br><br>(Piovesan et al., 2018) (Di Domenico et al., 2012)	375909

			(Piovesan et al., 2017) 22-08-2024 09:15:00	
PDBTot	Dataset of complexes containing IDRs; the IDR undergoes either a DOR or a DDR transition	-	(Horvath et al., 2020)<br><br>(Miskei et al., 2020)	513
PDBCDR	Dataset of complexes containing IDRs; the IDR undergoes both DOR and DDR transitions in a context-dependent manner	-	(Horvath et al., 2020)<br><br>(Miskei et al., 2020)	164
